# Uncovering molecular mechanisms of regulated cell death in the naked mole rat

**DOI:** 10.18632/aging.202577

**Published:** 2021-01-28

**Authors:** Alexei Evdokimov, Alexei Popov, Elena Ryabchikova, Olga Koval, Svetlana Romanenko, Vladimir Trifonov, Irina Petruseva, Inna Lavrik, Olga Lavrik

**Affiliations:** 1 Institute of Chemical Biology and Fundamental Medicine, Siberian Branch of the Russian Academy of Sciences, Novosibirsk 630090, Russia; 2 Institute of Molecular and Cellular Biology, Siberian Branch of the Russian Academy of Sciences, Novosibirsk 630090, Russia; 3 Translational Inflammation Research, Medical Faculty, Center of Dynamic Systems, Otto von Guericke University Magdeburg, Magdeburg 39106, Germany

**Keywords:** regulated cell death, naked mole rat, apoptosis, necrosis, DNA damage

## Abstract

The naked mole rat (NMR), *Heterocephalus glaber,* is the longest-living rodent species, and is extraordinarily resistant to cancer and aging-related diseases. The molecular basis for these unique phenotypic traits of the NMR is under extensive research. However, the role of regulated cell death (RCD) in the longevity and the protection from cancer in the NMR is still largely unknown. RCD is a mechanism restricting the proliferation of damaged or premalignant cells, which counteracts aging and oncotransformation. In this study, DNA damage-induced cell death in NMR fibroblasts was investigated in comparison to RCD in fibroblasts from *Mus musculus*. The effects of methyl methanesulfonate, 5-fluorouracil, and etoposide in both cell types were examined using contemporary cell death analyses. Skin fibroblasts from *Heterocephalus glaber* were found to be more resistant to the action of DNA damaging agents compared to fibroblasts from *Mus musculus*. Strikingly, our results revealed that NMR cells also exhibit a limited apoptotic response and seem to undergo regulated necrosis. Taken together, this study provides new insights into the mechanisms of cell death in NMR expanding our understanding of longevity, and it paves the way towards the development of innovative therapeutic approaches.

## INTRODUCTION

*Heterocephalus glaber (H. glaber)*, or the naked mole rat (NMR), is the longest-living rodent with a maximum lifespan of 32 years [1]. There are several ways of comparing longevity between various organisms and species. One of them is the comparison of the size and the lifespan across different animals [1]. According to this estimation, the lifespan of the NMR is almost ten times longer than the lifespan of the similarly sized rodent species *Mus musculus (M. musculus)*. The unique feature of the NMR is its protection from cancer [2, 3], with spontaneous tumors being extremely rare [4]. The NMR has evolutionarily developed a variety of adaptations that may contribute to its longevity and cancer resistance [5–10]. Some of these adaptations may promote genome and proteome stability and increase the resistance to stress. The comparison of activity of base excision repair (BER) and nucleotide excision repair (NER) in mouse and NMR fibroblasts allowed us to show that the NMR has more efficient excision repair systems than the mouse, which might contribute to longevity and cancer resistance of this species [11]. Moreover, the metabolic activity of fibroblasts from the NMR has been reported not to undergo strong changes upon paraquat and low-glucose media treatment, which is in contrast to mouse fibroblasts [12]. The adaptation of regulated cell death (RCD) mechanisms in the NMR might be directly linked to their resistance to cancer. However, in-depth studies of RCD in the NMR and its role in cancer development have not been conducted yet.

Apoptosis is a program of RCD, which is common to all multicellular organisms [13]. The apoptosis program is essential for development, the removal of dangerous cells and cellular homeostasis. Defects in apoptosis regulation lead to a number of malignancies such as cancer and autoimmune diseases. The key mediators of apoptotic cell death are caspases, the enzymes that mediate apoptosis initiation, as well as effector and demolition phases of apoptosis. Caspases are divided into initiator and effector caspases. Effector caspases-3 and -7 are main mediators of apoptosis that are directly involved in the proteolysis of cellular substrates and subsequent cell death. There are two major pathways of apoptosis induction: the extrinsic pathway and the intrinsic or mitochondrial pathway [14]. The latter is induced by a number of factors including DNA damage. Interestingly, recently, DNA damage has been also reported to induce regulated necrosis programs, in particular upon treatment with high doses of genotoxic stress agents [15, 16]. However, the ability of NMR cells to undergo cell death in response to DNA damage as well as molecular mechanisms of these processes have barely been studied.

DNA damage is a universal stress that all multicellular organisms have to counteract. Here, we compared the cell death responses to DNA damage in skin fibroblasts from *H. glaber* and *M. musculus*. Three compounds that are well known to induce three different types of DNA damage were used to trigger cell death: methyl methanesulfonate (MMS) [17], 5-fluorouracil (5FU) [18], and etoposide (Eto) [19]. It was demonstrated that skin fibroblasts from the NMR are less sensitive to DNA damage-induced cell death compared with mouse fibroblasts. This provides new insights into the mechanisms of response to DNA damage in the NMR and their longevity.

## RESULTS

### *M. musculus* fibroblasts are more sensitive to DNA damage compared to fibroblasts from H. glaber*H. glaber*

**Figure 1:**
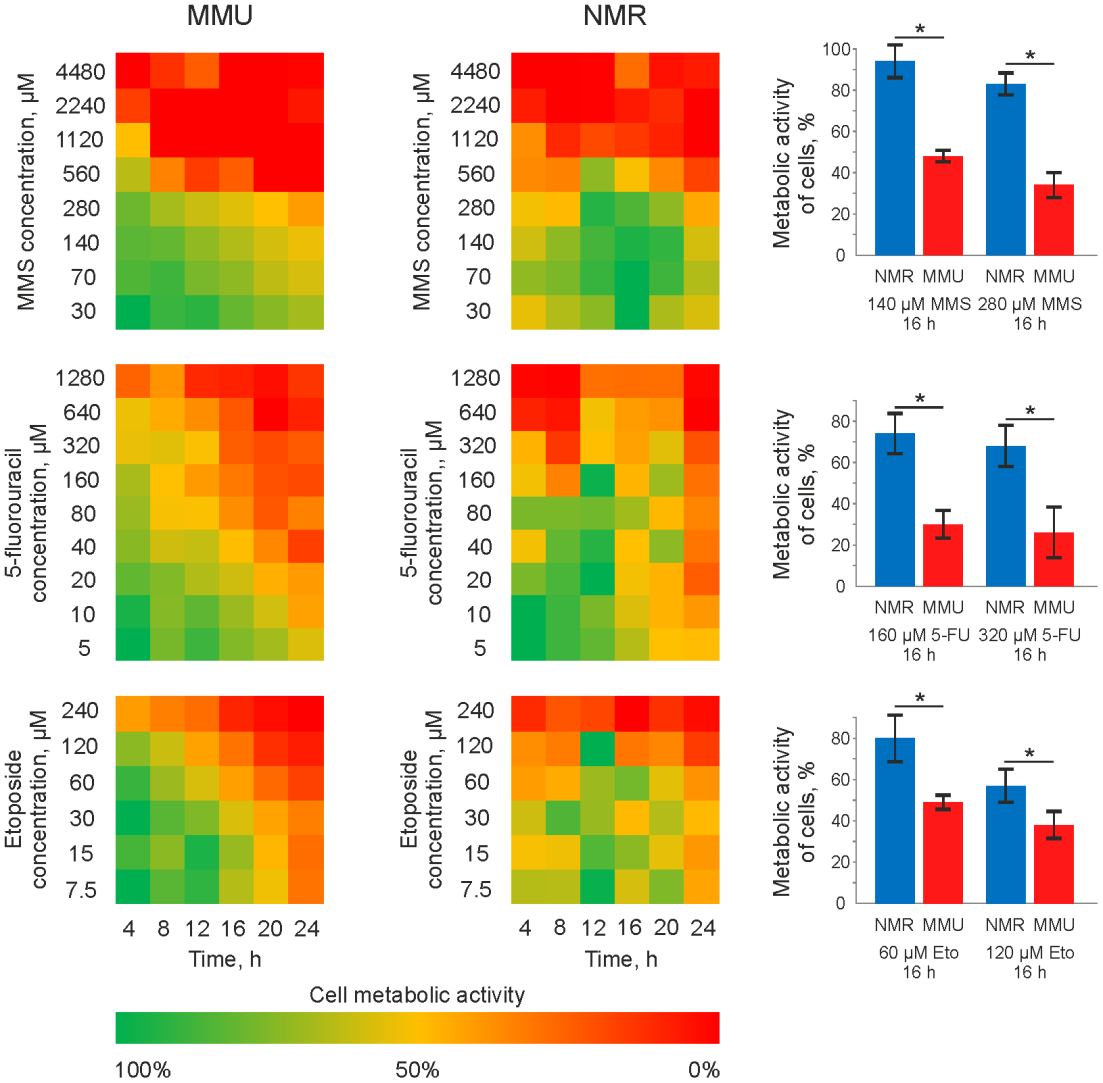
**Metabolic activity of mouse and NMR fibroblasts upon treatment with 5FU, MMS and Eto.** Mouse (MMU) and NMR fibroblasts, NSF8 and 3T3 cells, respectively, were treated with indicated DNA damaging agents. The conditions of treatment (time and concentrations of cytotoxic agents) are indicated on the plot. The color changing from green to red indicates the decrease in metabolic activity. The metabolic activity of untreated cells was taken as 100% and corresponds to the green color on the plot. Right side: a major decrease in metabolic activity, which was about 20-40%, is shown for the treatment of NMR fibroblasts with 280 μM of MMS, 320 μM of 5FU and 120 μM of Eto. These results are presented together with the decrease of metabolic activity in mouse fibroblasts upon treatment with the same concentrations of DNA damaging agents. Abbreviations: Mouse (MMU); 5-fluorouracil (5FU), etoposide (Eto), and methyl methanesulfonate (MMS). The standard deviation is shown; the confidence is based on the Mann-Whitney U test, *P < 0.05.

To compare the sensitivity of skin fibroblasts from *H. glaber* vs. *M. musculus* towards DNA damage, metabolic assays were carried out. For this comparative analysis, immortalized skin fibroblasts from both *H. glaber* and *M. musculus, e.g.* NSF8 and 3T3 cell lines, respectively, (termed thereafter NMR and mouse cells) were taken in the exponential growth phase (Supplementary Figure 1). NMR and mouse cells were treated in a time- and dose-dependent manner with MMS, 5FU and Eto (Figure 1). A notable decrease in the metabolic activity of NMR cells was detected at higher concentrations of MMS, 5FU and Eto compared to mouse cells. This was observed for all time intervals. Moreover, upon treatment with the same concentrations of MMS, Eto and 5FU, a longer exposure time was needed to reduce the metabolic activity of NMR cells in comparison to mouse cells (Figure 1). It has to be noted that the concentrations of MMS, 5FU and Eto, that are commonly used in the studies of DNA damage-induced apoptosis, are in the range between 10 and 70 μM. Indeed, a notable metabolic activity loss of mouse fibroblasts was observed upon treatment with 10 to 70 μM MMS, 5FU and Eto (Figure 1). However, incubation using the same concentration range of DNA damaging agents, led to an only marginal decrease of the metabolic activity in the case of NMR fibroblasts. Moreover, the drop of metabolic activity of NMR fibroblasts was observed starting from the concentration of 100 μM only. In particular, a strong decrease in metabolic activity (20-40%) was observed in 16 hours for the treatment of NMR fibroblasts with 280 μM of MMS, 320 μM of 5FU and 120 μM of Eto (Figure 1, right side). Taken together, we can conclude that *M. musculus* fibroblasts are more sensitive to DNA damage-induced metabolic activity loss compared to fibroblasts from *H. glaber*.

### *M. musculus*fibroblasts are characterized by a higher caspase activity upon treatment with DNA damage agents compared to fibroblasts from *H. glaber*

**Figure 2:**
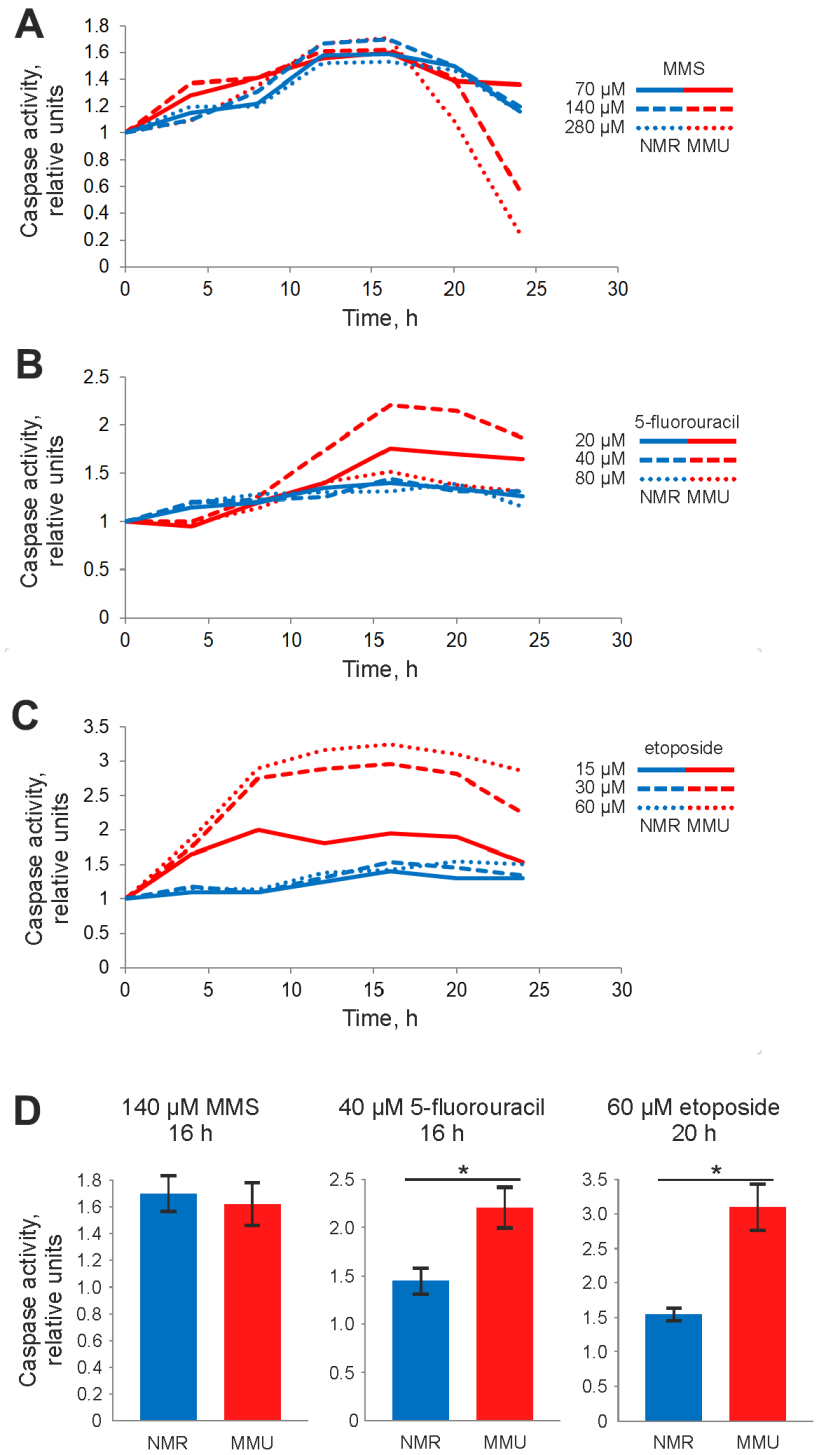
**Caspase activity of NMR and mouse fibroblasts upon treatment with DNA damaging agents determined by Caspase-Glo 3/7 assay.** Activity of effector caspases-3/7 was measured after treatment with: (**A**) 70 μM (solid line), 140 μM (dashed line), and 280 μM (dotted line) MMS; (**B**) 20 μM (solid line), 40 μM (dashed line) and 80 μM (dotted line) 5FU; (**C**) 15 μM (solid line), 30 μM (dashed line), and 60 μM (dotted line) Eto. The caspase activity in untreated cells was taken as one relative unit. Results represent the mean of at least three independent experiments. (**D**) Caspase activity of NMR and mouse cells is shown for treatment with 140 μM MMS for 16 h, 40 μM 5FU for 20 h and 60 μM Eto for 16 h. Standard deviation is shown; confidence is based on the Mann-Whitney U test, *P < 0.05. The activity of NMR and mouse cells is shown in blue and red, respectively. Abbreviations: Mouse (MMU); 5-fluorouracil (5FU), etoposide (Eto), and methyl methanesulfonate (MMS).

The differences in metabolic response to DNA damage allowed suggesting that *H. glaber* and *M. musculus* might have a different sensitivity to DNA damage-induced cell death. Activation of effector caspases-3 and -7 is a hallmark of apoptosis. To examine effector caspase activation in fibroblasts from *M. musculus vs*. *H. glaber* caspase-3/7 activity was measured in a time- and dose-dependent manner. For mouse fibroblasts, we selected concentrations of MMS, 5FU and Eto that cause strong changes in metabolic activity (Figure 1). Treatment with these concentrations of all three DNA damaging agents caused an increase of caspase-3/7 activity in mouse fibroblasts. The maximal value of caspase activity was reached in 16 h after addition of MMS and 5FU (Figure 2A, 2B), and from 16h to 20 h for Eto treatment (Figure 2A–2C). However, the observed increases in caspase activity were not very high: 1,6 fold for MMS, 2-fold for 5FU and 3-fold for Eto (Figure 2D). Moreover, the maximal values of caspase activity were relatively robust towards the dose of DNA damaging agent. This indicates that mouse fibroblasts show only moderate levels of caspase-3/7 activity upon incubation with MMS, 5FU and Eto.

To compare caspase activation in fibroblasts from *H. glauber vs. M. musculus* the same concentrations of MMS, 5FU and Eto were used for NMR cells. In line with the observed lower sensitivity in metabolic response, fibroblasts from *H. glaber* showed lower levels of caspase activity compared to fibroblasts from *M. musculus*. Only a small induction of caspase-3/7 activity was detected upon MMS treatment of NMR cells, while almost no increase of caspase activity was observed upon Eto and 5FU treatment (Figure 2A–2D). In particular, upon treatment with 70 μM MMS, the increase in caspase activity was observed already at 8 hours after MMS treatment, peaking at 16 hours. A similar dependence was observed upon higher concentrations of MMS (Figure 2A). Interestingly, the same time dependence was also found for 5FU and Eto treatments, though caspase-3/7 activity was rather weak within the selected concentration range of 5FU and Eto.

Collectively, the DNA damage-induced caspase activity of fibroblasts from *H. glaber* was much lower than that of *M. musculus*. Next we aimed to compare the cell death responses of *H. glaber vs. M. musculus* fibroblasts upon MMS, 5FU and Eto treatment.

### Flow cytometry indicates a higher rate of cell death in fibroblasts from *M. musculus* *vs* *H. glaber*

The cell death induction in fibroblasts from *M. musculus vs H. glaber* was compared using flow cytometry analysis with FITC-AnnexinV and propidium iodide (PI) staining. The measurements were performed at the time point of maximal caspase activity assuming that under these conditions cells show a strong apoptotic response. Accordingly, NMR and mouse cells were exposed to Eto as well as 5FU for 20 h; and 5FU for 16 h. The relative number of viable (AnnexinV^-^/PI^-^), apoptotic (AnnexinV^+^/PI^-^) and late apoptotic/necrotic (AnnexinV^+^/PI^+^) cells was then measured. Importantly, we assumed that a population of double positive AnnexinV^+^/PI^+^ cells might comprise both late apoptotic or secondary necrotic cells as well as cells that undergo regulated necrosis [20]. The latter is based on reports that DNA damage might induce the regulated type of necrosis, necroptosis, in particular upon high concentrations of DNA damaging agents [21].

**Figure 3:**
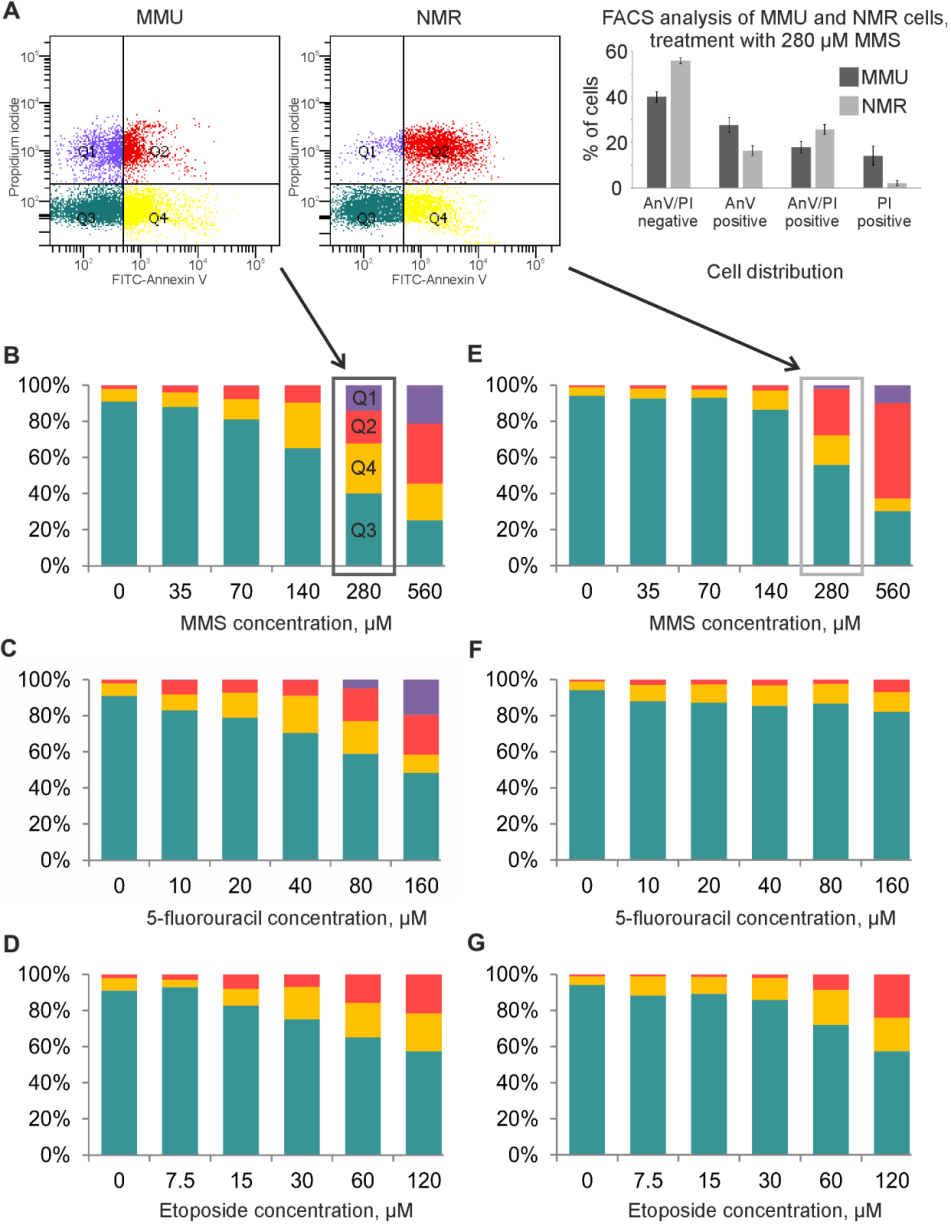
**Flow cytometry analysis of MMU and NMR cells upon treatment with DNA damaging agents.** (**A**). NMR and mouse cells were stained with FITC-Annexin V (AnnV) and Propidium Iodide (PI). The gating strategy for double negative AnnexinV^-^/PI^-^(Q3), single positive, early apoptotic cells (AnnexinV^+^/PI^-^) (Q4), single positive (AnnexinV^-^/PI^+^) (Q1) and double positive AnnexinV^+^/PI^+^ (Q2) is shown. (**B**–**G**). Cell death of MMU (**B**–**D**) and NMR (**E**–**G**) cells under the wide spectrum of cytotoxic conditions was evaluated by FACS analysis. The total number of cells was taken as 100%; blue color indicates the population of viable cells, double negative AnnexinV^-^/PI^-^(Q3); yellow color is used for single positive, early apoptotic cells (AnnexinV^+^/PI^-^) cells (Q4); red color is implemented for double positive AnnexinV^+^/PI^+^ cells (Q2); purple color for single positive (AnnexinV^-^/PI^+^) cells (Q1). The representative experiment out of three independent ones is shown.

In accordance with the results from caspase activity and metabolic assays, NMR cells turned out to be more resistant towards DNA damage-induced cell death compared to mouse cells. In NMR cells, the relative number of viable cells decreased at higher concentrations of reagents compared with mouse cells (Figure 3). Furthermore, early apoptotic cells (AnnexinV^+^/PI^-^) and double positive AnnexinV^+^/PI^+^ cells were detected upon treatment with higher concentrations of DNA damaging agents in NMR cells than in mouse cells. In particular, strong differences were observed upon 5FU treatment, where NMR cells remained largely resistant (Figure 3). Differences were also observed upon treatment with MMS and Eto. In particular, strong differences were monitored upon incubation with 70 and 140 μM of MMS as well as 15 and 30 μM of Eto. Importantly, treatment with all three DNA damaging agents led to the detection of a population of early apoptotic cells (AnnexinV^+^/PI^-^) indicating that NMR cells undergo apoptosis. Though, as mentioned above, this population was much smaller in NMR cells compared to mouse cells treated with the same concentration of DNA damaging agents. Upon increase of the concentration of DNA damaging agents, a population of double positive AnnexinV^+^/PI^+^ cells were observed. As mentioned above, we assumed that this population might comprise both late apoptotic and necroptotic cells.

To examine whether high concentrations of DNA damaging agents induce the necroptotic type of cell death in NMR cells, we applied a pharmacological inhibitor of necroptosis (nec-1). Nec-1 inhibited the loss of metabolic activity induced by 280 μM of MMS (Supplementary Figure 2). These results indicate that high concentrations of DNA damaging agents induce the regulated necrotic pathway. Taken together, we have shown that MMS, Eto and 5FU induce apoptotic and likely necrotic types of cell death in NMR cells. Moreover, NMR cells were less sensitive towards DNA damage-induced cell death compared to mouse cells.

### Electron microscopy supports the apoptotic and necrotic phenotype of cell death in fibroblasts from *M. musculus* and *H. glaber*

**Figure 4:**
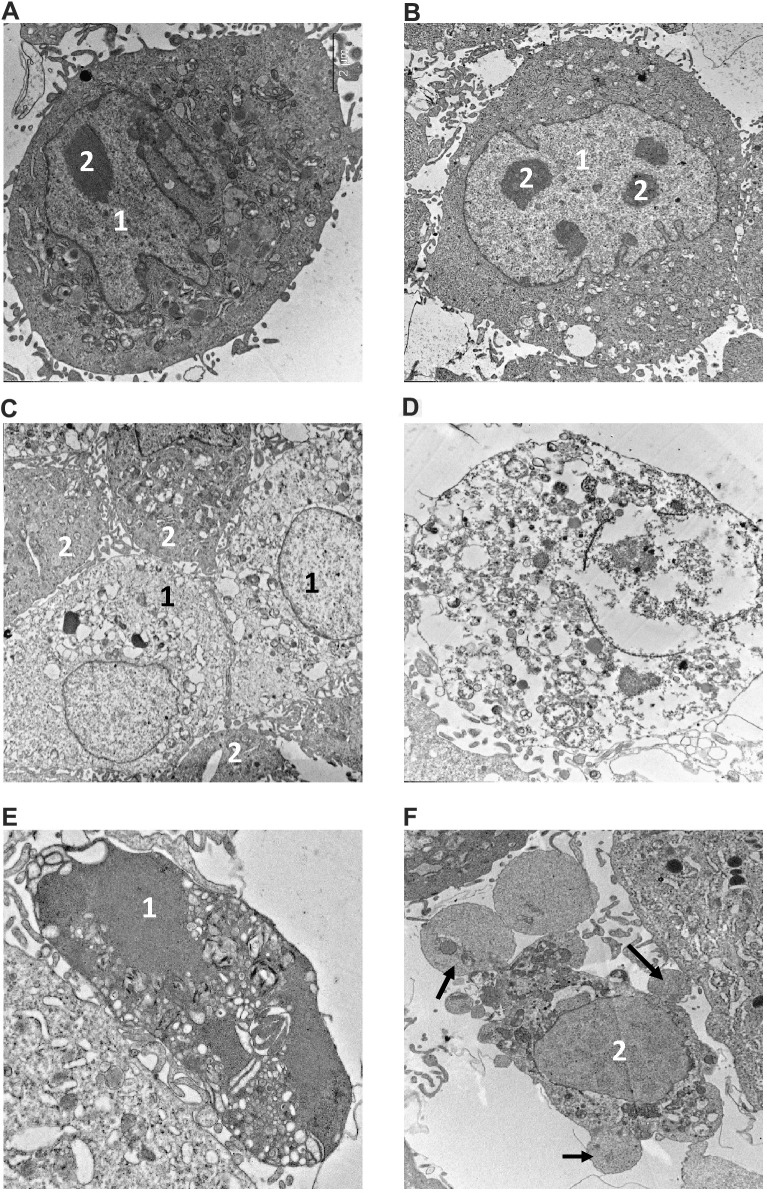
**Electron microscopy analysis of MMU and NMR cells upon treatment with MMS.** (**A**, **B**). The images of cells from NMR and mouse, that remained untreated, are shown. 1 – nucleus, 2 – nucleolus. (**C**–**F**). The images of cells after incubation with MMS. (**C**) swollen NMR cells (1) with cytoplasm of low electron density; and cells without signs of alteration (2) are shown. (**D**) necrotic mouse cell is shown, in which organelles are completely destroyed. Apoptosis of NMR (**E**) and mouse (**F**) cells. NMR cell nucleus (1) with condensed chromatin and organelles is shown, mouse cell demonstrates extensive “apoptotic blebbing” (“apoptotic blebs” are shown with arrows), 2 – nucleus without chromatin condensation but altered morphology.

The morphology of dying cells is a key feature indicating the type of cell death. We performed electron microscopy (EM) analysis of NMR and mouse fibroblasts treated with toxic agents to verify the presence of different morphological features of cell death. The ultrastructure of both NMR and mouse cells in control samples (Figure 4A, 4B) did not show any signs of destruction. In particular, the cytoplasm with average electron density contained a well-developed endoplasmic reticulum and other organelles. Large nuclei in NMR cells were more indented in comparison to those in mouse cells. The nuclei of both cell kinds contained several nucleoli. Control samples of NMR and mouse cells contained very few necrotic cells (0-1 per 100 cells).

Incubation of NMR and mouse cells with 280 μM of MMS during 16 h resulted in a number of swollen cells in both cell types, which might represent the first step towards necrosis (Figure 4C). However, the plasmalemma of these cells had retained its integrity, and therefore the state of these cells could not be attributed to necrosis, but rather to late apoptosis. In addition, a number of necrotic cells with disrupted plasmalemma, nucleus and other organelles were observed (Figure 4D). The ultrastructure of necrotic cells did not differ in NMR and mouse cells, as in the case of apoptosis, the signs of which were observed in both cell types incubated with MMS. This indicated that these stimulation conditions led to the induction of both types of cell death: apoptosis and regulated necrosis. Furthermore, it has to be noted that examining the samples after 16 hours of treatment, we observed the cells at different stages of apoptosis. This explains differences in apoptotic cells observed in ultrathin section, in particular, in morphology of the nucleus and the presence of apoptotic blebbing (Figure 4E, 4F). The obtained data were in good agreement with flow cytometry analysis and observations that upon incubation with 280 μM of MMS for 16 h, the majority of cells in both NMR and mouse fibroblasts are double positive (Annexin V^+^/PI^+^). The latter population might contain both late apoptotic or secondary necrotic as well as necroptotic cells, which is in good agreement with the EM analysis.

**Figure 5:**
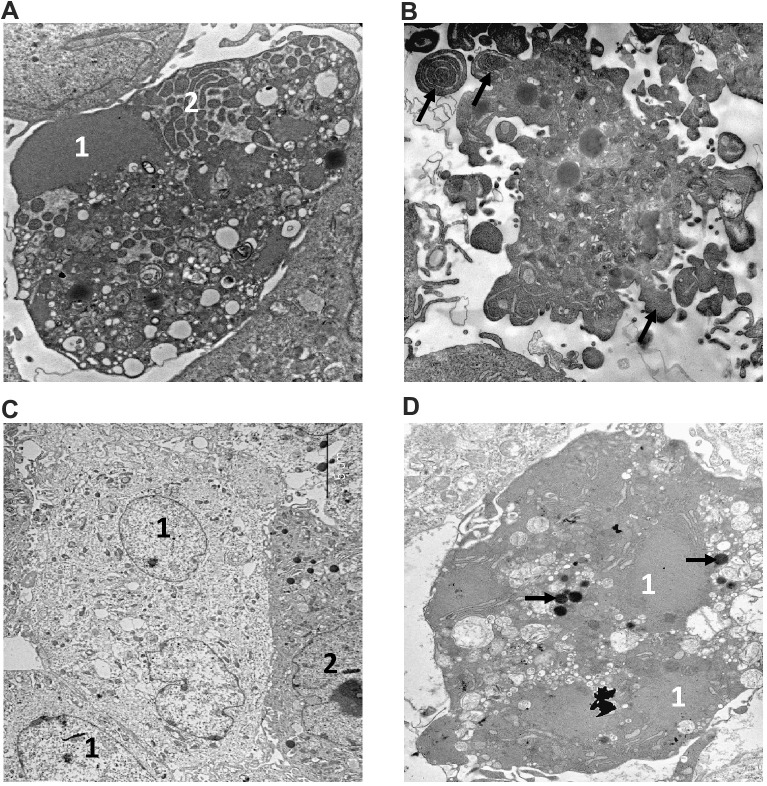
**Electron microscopy analysis of MMU and NMR cells upon treatment with 5FU and Eto.** Apoptotic features of cells from NMR (**A**) and mouse (**B**) incubated with 160 μM of 5FU are shown. The NMR cell is filled with the remnants of the nucleus (1) with condensed chromatin and organelles; the endoplasmic reticulum area is marked with the number "2". (**B**) section of mouse cell periphery demonstrates extensive apoptotic blebbing” (“apoptotic blebs” are shown with arrows). (**C**, **D**). The images of NMR cells treated with Eto are shown. (**C**) swollen cells (1) with cytoplasm of low electron density, and cell without signs of alteration (2). (**D**) apoptotic cell with a highly condensed nucleus (1) and cytoplasm. Organelles are not seen; arrows show lipid droplets.

Examination of mouse cells incubated with 160 μM of 5FU for 16 h revealed many destroyed cells with morphologic signs of necrosis (3-4.5 per 100 nucleus containing cells), while necrotic cells in NMR samples were rare (0.5-1 per 100 cells). Cells with signs of apoptosis (Figure 5A, 5B) were relatively rare in both NMR and mouse samples. This was in accordance with the flow cytometry analysis, in which a lower sensitivity of NMR cells towards 5FU had been detected. Moreover, these data fit well to the suggestion that upon high concentrations of DNA damaging agents, cells rather undergo regulated necrosis, which was observed in these experiments.

For EM analysis with Eto, we took the lower concentration of Eto to examine the initial steps of cell death induction in NMR and mouse fibroblasts. Cells of NMR and mouse samples under Eto (60 μM) treatment for 16 h mostly did not show visible signs of destruction, however, some NMR cells were observed to swell. Moreover, cytoplasm and nucleus had less electron density than untreated cells (Figure 5C). Necrotic cells were not numerous in either NMR or mouse samples treated with Eto (0.8-1.0 per 100 cells). A similar frequency was observed with respect to apoptotic cells (Figure 5D). These results are in accordance with the data obtained with flow cytometry, which indicated a very low number of dying cells with this concentration of Eto and the induction of both cell death pathways.

Thus, electron microscopic examination of ultrathin sections of NMR and mouse cells incubated with MMS, 5FU, and Eto revealed a correspondence of the morphological characteristics of the cell samples to those established by flow cytometry analysis. These analyses have demonstrated that both pathways, apoptosis and regulated necrosis, are induced upon MMS, 5FU, and Eto treatment, which is in the good agreement with caspase activity and flow cytometry assays.

## DISCUSSION

The current study addressed the response of NMR skin fibroblasts to DNA damage, which is a universal stress that all organisms have to counteract. We have shown that treatment with DNA damaging agents induces cell death in NMR skin fibroblasts, which involves both apoptotic and likely necrotic RCD programs. In our study, three genotoxic agents with different mechanisms of action were employed. All three of them caused cell viability loss and appearance of the markers of cell death. Interestingly, fibroblasts from mouse cells showed a higher sensitivity to DNA damage-induced cell death compared to fibroblasts from NMR.

NMR cells underwent cell death upon higher concentrations of DNA damaging agents compared to mouse cells. High concentrations of DNA damaging agents are described to trigger the programs of regulated necrosis in mice and humans such as necroptosis [21, 22]. Accordingly, we assumed that similar mechanisms might be involved in NMR cells. Of note, nec-1, a pharmacological inhibitor of the necroptosis pathway [23], showed protection from the effects of DNA damaging agents on NMR cells. Moreover, cells with the features of regulated necrosis were detected among MMS- and 5FU-treated NMR cells using EM. Finally, flow cytometry analysis of the NMR cells treated with the high dose of DNA damaging agents revealed the appearance of a population of double positive cells that might comprise necrotic cells in addition to late apoptotic cells. These results allow assuming that NMR cells undergo regulated necrosis. However, we do not exclude the induction of apoptosis as early apoptotic cells were detected by flow cytometry assays, while late apoptotic cells were detected by EM. The induction of both apoptosis and necrosis pathways upon DNA damage has been well documented [16]. The intricate molecular balance of apoptosis and regulated necrosis in NMR cells has to be addressed in future studies.

There might be several reasons for the different response of NMR cells *vs* mouse cells. The first one would involve the higher threshold for switching to the cell death response upon DNA damage compared to mouse cells. Indeed, we have shown in previous studies that DNA repair systems are more efficient in NMR fibroblasts compared to mouse cells [11]. This would suggest that a highly efficient DNA repair system protects this organism from cell death initiation. Another reason might include a different lipid composition of the extracellular membrane of NMR *vs* mouse cells, resulting in a different rate of transport of chemotherapeutic drugs into the cells. This has to be addressed in future studies. Yet another important reason might involve differences in molecular mechanisms of apoptosis and necroptosis in NMR *vs* mouse cells, which includes a potential major disbalance of the apoptotic network in NMR cells. Indeed, cell death-inducing complexes and their inhibitors are barely studied in NMR cells and have to be investigated in the future. Finally, the differences in cell death response might be due to different intracellular turnover products of 5FU, MMS and Eto in these two cell types as discussed below.

5FU is an analogue of uracil, in which the hydrogen atom at position 5 is replaced with fluorine. In cells, 5-fluorouracil undergoes a metabolic conversion into fluorodeoxyuridine monophosphate (FdUMP), a suicide substrate for thymidylate synthase [18]. As a result, the deoxythymidine monophosphate (dTMP) pool depletes. Another action of this reagent is associated with further conversion of FdUMP into FdUTP and incorporation of FdUMP into DNA during replication. The accumulation of such modified bases in DNA chains may cause cell death. Moreover, fluorouridine monophosphate could also be incorporated in RNA during transcription, leading to RNA stress and apoptosis [24]. Our experiments demonstrated that exposure to 5FU results in apoptosis and regulated necrosis in mouse cells. The metabolic activity of NMR cells treated with this reagent decreased as well as the appearance of early apoptotic cells and double positive cells, although the response towards 5FU was really low in NMR cells. It was assumed that genome maintenance mechanisms of the NMR could protect its cells against the action of 5-fluorouracil, at least at the doses used in this study. It is also possible that the peculiarities of the 5-fluorouracil metabolism in NMR cells result in decreased synthesis of FdUMP, FdUTP and FUTP. Meanwhile, the decline in metabolic activity of NMR cells after exposure to 5-fluorouracil (Figure 1) is likely to correspond to a reduction of their proliferative activity. The inhibition of proliferation and cell cycle arrest could be beneficial as they give cells additional time for DNA repair and prevent the replication of DNA with incorporated fluorodeoxyuridine.

MMS is a typical methylating agent with a well-studied mechanism of action [17]. MMS can act as an alkylating agent and cause single-point mutations [25]. MMS can stall a replication fork at the sites of DNA cross-links in dividing cells, thus causing DNA double-strand breaks [26]. Double-strand breaks are among the most severe DNA damages. If they fail to be processed by the cellular DNA repair system, the formation of DNA double-strand breaks can lead to cell cycle arrest and apoptosis [27]. Methylated nucleobases are the substrates of cellular DNA repair systems (e.g., BER). NMR cells were shown to exhibit high resistance to such reagents compared to mouse cells. This difference was expected, since NMR cells display more efficient DNA excision repair than mouse cells do [11]. Accordingly, it might be suggested that NMR cells trigger apoptosis upon much higher concentrations of MMS than mouse cells as the DNA damage from lower concentrations of MMS is efficiently counteracted by the DNA repair systems of the NMR.

The third reagent used in our study is Eto, which is a topoisomerase II inhibitor. This compound binds to topoisomerase II and prevents DNA religation, causing the formation of a large amount of DNA breaks in dividing cells [19]. The conclusion can be drawn from our findings that Eto induces an apoptotic response in both mouse and NMR cells. However, a smaller decline in metabolic activity is observed in NMR cells (Figure 1). Moreover, the relative number of viable NMR cells decreases at significantly higher reagent concentrations compared to mouse cells (Figure 3). It is fair to assume that the differences in responses of NMR and mouse cells to the exposure of DNA damaging agents correspond to a higher efficiency of DNA repair systems in NMR cells in comparison to mouse cells and, on the other hand, to the limited efficiency of proapoptotic cascade activation and limited apoptotic response of NMR cells.

The features of regulated NMR cell death observed in this study may represent alternations in the chain of molecular events preceding apoptosis. It was shown previously that NMR cells are resistant to the proapoptotic effects of H_2_O_2_ [28]. Furthermore, long-living organisms have relatively low expression levels of apoptosis-related genes [29]. Taking into consideration the high activity of DNA repair systems in NMR [11], we suggest that the limited apoptotic response of NMR cells could play an important role in longevity as it leaves sufficient time for DNA repair to occur.

## CONCLUSIONS

Our comparative study revealed that NMR cells are highly resistant to all types of exposure of DNA to DNA damaging agents. The decline in cellular metabolic activity and induction of cell death take place at much higher concentrations of cytotoxic agents in NMR cells compared to mouse cells. Unlike mouse cells, NMR cells are especially resistant to several proapoptotic reagents (e.g., 5FU as it was demonstrated in this study). Our data, obtained using various approaches such as flow cytometry, electron microscopy and caspase activity assays, give ground for the assumption that the efficiency of apoptosis activation in the NMR is lower compared to that in the mouse. When exposed to high-dose toxic agents, NMR cells might undergo necrotic death. The molecular balance between these two types of cell death in the NMR is an important subject of future research.

## MATERIALS AND METHODS

### Cell culture

The establishment of cell cultures derived from NMR fibroblasts was described earlier [7]. Immortalized NMR skin fibroblasts, NSF8, were kindly provided by Dr. V. Gorbunova (University of Rochester, Department of Biology). NMR cells were cultured in MEM supplemented with 15% FBS (Gibco), 10% AmnioMAX II Complete Medium (Gibco), 5 ng/ml bFGF, 10^5^ U/L penicillin, 100 mg/L streptomycin, and 2.5 mg/L amphotericin β at 32° C in the presence of 5% CO_2_. The cell line was deposited in the cell bank of the Institute of Molecular and Cellular Biology, SB RAS (“The General Collection of Cell Cultures”, No. 0310-2016-0002).

The 3T3 mouse cell line was kindly provided by Dr. V. Fishman (Sector of Genomic Mechanisms of Ontogenesis, Institute of Cytology and Genetics, Russia). Cells were cultured in MEM supplemented with 15% FBS (Gibco), 10^5^ U/L penicillin, 100 mg/L streptomycin, and 2.5 mg/L amphotericin β at 37° C in the presence of 5% CO_2_.

All experiments were carried out with dividing cells in the exponential growth phase. Cell growth curves (Supplementary Figure 1) were used to determine the parameters of cell growth.

### Metabolic assay

Cells were seeded in 96-well plates at a concentration of 3 × 10^3^ cells/well in 200 μl culture medium and cultured as described above for 24 (NSF8 cells: NMR fibroblasts) or 12 (3T3 cells: Mouse fibroblasts (MMU)) h. Afterwards, the compounds dissolved in DMSO were added, and the cells were incubated for various time intervals. The EZ4U test (Biomedica) was carried out according to the manufacturer’s instructions. Briefly, 20 μl of EZ4U labeling mixture was added, and the cells were incubated for 2–3 h. Absorbance of the samples was measured at 492 nm using a microplate reader CLARIOstar Plus (BMG LABTECH GmbH). All experiments were repeated in the triplicate.

### Apoptosis detection

NMR (NSF8 cells) and MMU (3T3 cells) cells were seeded in 6-well plates and cultured as described above. When the cell cultures reached 80% confluence, the cells were treated with the DNA damaging compounds for 16 (5FU) or 20 (Eto and MMS) h. Before treatment with trypsin, detached cells were collected. Adherent cells were rinsed with PBS and detached from the plate with trypsin. Soybean trypsin inhibitor (Sigma) was added up to 10 μg/ml concentration to inhibit trypsin-initiated proteolysis. The detached and trypsinized cells were combined and pelleted by centrifugation (5 min at 500 g). FITC-Annexin V Apoptosis detection kit and FACSCanto II (BD Biosciences, San Jose, USA) flow cytometer were used to detect cell death. The analysis was performed on a FACSCantoII flow cytometer (Becton Dickinson) using the FACSDiva Software (BD Biosciences). Cells were initially gated based on forward scatter *vs.* side scatter to exclude small debris. Ten thousand events from each population were collected. The inhibitors nec-1 (Sigma-Aldrich, USA) and z-VAD-fmk (BD Biosciences) dissolved in DMSO were added 2 hours before addition of DNA damaging reagents.

### Caspase activity detection

Cells were seeded in 96-well plates at a concentration of 3 × 10^3^ cells/well in 200 μl culture medium and cultured as described above to reach 60% confluence. Caspase activity was measured using the Caspase-Glo 3/7 assay kit according to the manufacturer's instructions (Promega, Madison, WI). Briefly, 200 μl of Caspase-Glo 3/7 reagent was added to each well; the plate was shaken gently for 30 s and incubated at room temperature for 2 h. Luminescence of the samples was measured using an Infinite M200 plate reader (Tecan, Research Triangle Park, USA). The proluminescent substrate containing DEVD tetrapeptide was used. This substrate is capable of being cleaved by caspase-3/7 resulting in a release of aminoluciferin, a luciferase substrate. The activity of effector caspases-3/7 can be estimated according to luminescence intensity.

### Electron microscopy

NSF8 and 3T3 cell pellets were fixed after corresponding treatment in 4% paraformaldehyde and post-fixed in 1% osmium tetroxide solution, then routinely processed and embedded in Epon–Araldite mixture (SPI, USA). Semithin sections were prepared from hard blocks, stained with Azur 2, and examined by light microscopy to select the areas for ultrathin sectioning. Ultrathin and semithin sections were cut using an UC7 ultramicrotome (Leica, Germany), contrasted using uranyl acetate and lead citrate (SPI, USA), and examined using a JEM 1400 transmission electron microscope (Jeol, Japan). The images were collected using a side-mounted Veleta digital camera (EM SIS, Germany).

### Statistical analysis

Statistical analysis was performed using Statistica 10 software. All experiments were performed at least in triplicate. In the figures, bars show the standard deviation. The level of confidence was estimated using the Mann–Whitney *U* test.

## Supplementary Material

Supplementary Figures
