# Supplementary material for: Uncovering molecular mechanisms of regulated cell death in the naked mole rat
Source: Aging (Albany NY). 2021 Jan 28;13(3):3239–53. doi: 10.18632/aging.202577 (PMC7906159; doi:10.18632/aging.202577)
Supplement: Supplementary Figures [file aging-13-202577-s001.pdf]

SUPPLEMENTARY FIGURES

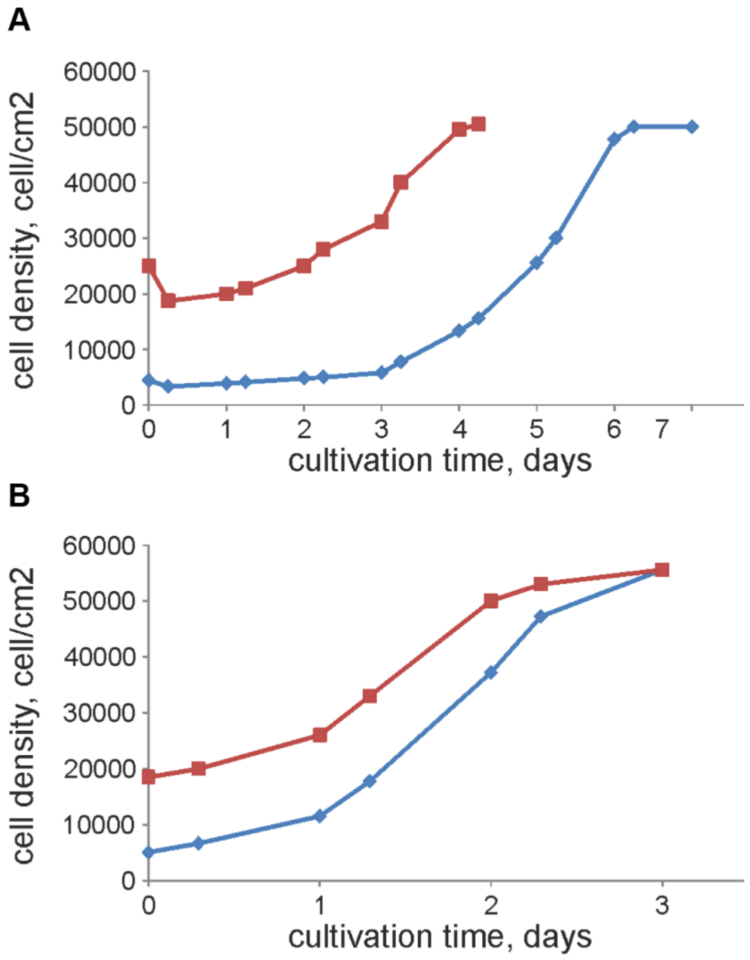

Supplementary Figure 1. Cell growth curves of NMR (**A**) and MMU (**B**) cells. The growth rate of NSF8 cells is significantly lower than that of 3T3 cells. Moreover, NMR cells have an additional mechanism of contact inhibition (early contact inhibition [6]), which prevents cells from reaching high density during cultivation. Since the properties of dividing and non-dividing cells are different, the growth curves (Supplementary Figure 1) were used to design the cultivation protocol, which allows to perform the experiments with extensively dividing cells in the exponential growth phase. NSF8 cell density corresponding to the exponential growth phase was determined to be 25,000 cells/cm2; while the corresponding exponential growth phase for 3T3 cells was observed at the cell density of 30,000 cells/cm2. These concentrations of cells were used in the experiments. To build the cell growth curves, cells were cultured on 6-well plates to maximal confluence and then were transplanted with 1/3 dilution for mouse cells and 1/2 dilution for NMR cells (red line). In another experiment, cells were diluted to 45,000 cells per well (blue line). The number of cells was counted twice a day.

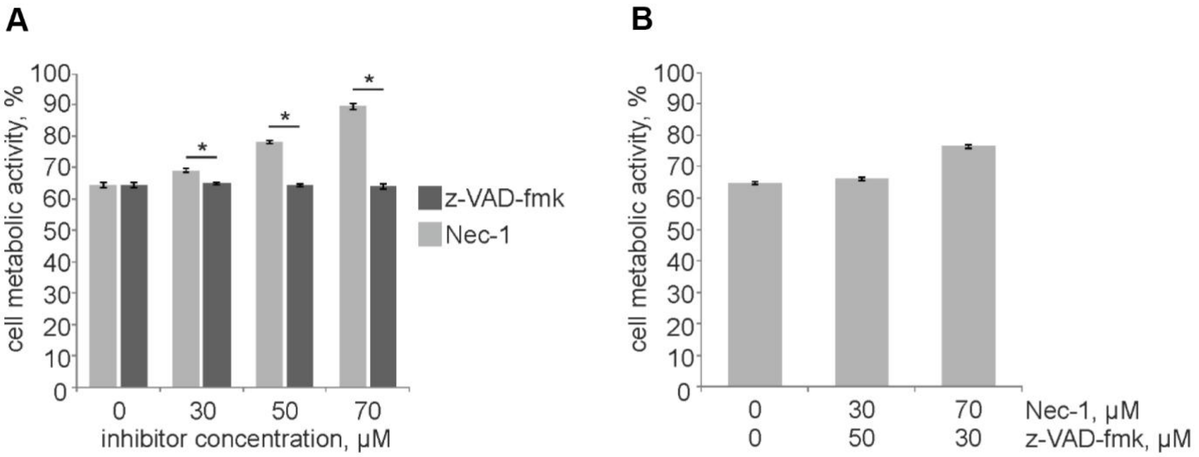

Supplementary Figure 2. Metabolic activity of NMR cells after treatment with 280 μM MMS in the presence of either Nec-1 or z-VAD-fmk (**A**) and co-treatment with 280 μM MMS and both inhibitors (**B**). Standard deviation is shown; confidence is based on the Mann-Whitney U test, *$P < 0.05$.
